# The $^{1}\!S_{0}$ channel of proton–proton scattering in new chiral effective field theory power counting

**DOI:** 10.1038/s41598-023-33735-6

**Published:** 2023-04-29

**Authors:** B. Behzadmoghaddam, M. Radin, S. Bayegan

**Affiliations:** 1 Department of Physics, University of Tehran, P.O. Box 14395-547, Tehran, Iran; 2 Department of Physics, K. N. Toosi University of Technology, P.O. Box 163151618, Tehran, Iran

**Keywords:** Mathematics and computing, Physics

## Abstract

In this paper, the chiral effective field theory is used to investigate the spin singlet channel $^{1\!}S_{0}$, of proton-proton (*pp*) scattering according to the new suggested power counting. For this purpose, the *pp* zero scattering amplitude is reproduced by inserting one pion exchange at leading order (LO) and the Coulomb interaction between protons at next-to-leading order (NLO). This leads to a systematic improvement up to NLO compared to the result obtained from Nijm93 potential model.

## Introduction

The nuclear effective field theory (EFT) technique is employed to extract the low-energy physics with only the relevant degrees of freedom and the operators. This provides a systematic and model-independent formalism to the problem of nuclear forces which is consistent with the low-energy symmetries of quantum chromodynamics (QCD)^1,2^. Therefore, the nuclear chiral EFT ($\chi$EFT) Lagrangian is written in terms of nucleons, pions and other hadron fields instead of the underlying particles such as quarks and gluons of QCD^3^.

The nuclear EFT for the description of the nucleon–nucleon (*NN*) systems offers a way systematically calculates low energy physical quantities up to a certain order. This systematic approach is based upon an organizing rule known as power counting (PC). Once specified, the low energy observables can be calculated order by order as an expansion in powers of the small parameter $Q/M_{hi}$, where *Q* is the characteristic external momentum of a process and $M_{hi}\!\lesssim \! M_{QCD}\sim$ 1 GeV is the EFT breakdown scale. In the *NN* systems, the spin singlet channel $^{1}\!S_{0}$, is a particular channel and has a very shallow virtual bound state with binding momentum $\aleph \sim$10 MeV which can be described by the nuclear EFT at the low energy limit successfully^4,5^.

In the early 90s, a PC scheme was proposed for nuclear $\chi$EFT by Weinberg^6^. In Weinberg’s PC (WPC) one determines the sum of irreducible diagrams in the expansion based on naive dimensional analysis (NDA) as the effective potential. Then the effective potential is used to solve the Lippmann–Schwinger (LS) or Schr$\ddot{\text {o}}$dinger equation. Although conceptually attractive and simple in numerical implementation, the divergences that arise in the leading order (LO) calculations of the *NN* amplitude on the basis of NDA cannot be absorbed by the LO operators in certain partial waves. Consequently, it fails to produce the *NN* amplitude at LO consistent with the renormalization group (RG) invariance in the $^{1}\!S_{0}$ channel at momenta of the order of pion mass $Q\sim m_{\pi }$^7^−^18^.

The other PC scheme suggested by Kaplan, Savage and Wise (KSW) was a consistent scheme with RG^7,19^. In the expansion developed by KSW only the contact interactions are treated non-perturbatively at LO, while others are treated perturbatively as subleading corrections. The KSW PC has been implemented for computing properties of the *NN* interactions in the $^{1}\!S_{0}$ channel in terms of an $\chi$EFT at low energies^7,20^. Though the KSW PC is a scheme consistent with RG, critical problems have been reported in description of the certain partial waves, such that the obtained *NN* scattering amplitudes at higher orders, shows no convergence in the low momenta^21^.

Besides the problems mentioned above according to the $^{1\!}S_{0}$ empirical phase shift, the zero amplitude at a center of mass (CM) momentum $k=k_{0}\simeq$ 340 MeV in both PC schemes has not been reproduced at the LO, compared to the Nijmegen partial wave analysis^22^. Recently, a new PC has been proposed in Ref.^23^ that has been provided the amplitude zero at LO, in addition to the shallow virtual state.

In this work, we continue our previous study^24^ with the aim of investigation of *pp* scattering through the *pp* $^{1}S_{0}$ amplitude obtained on the basis of the new suggested PC in the $\chi$EFT scheme up to NLO. Such an approach provides a new rearrangement of the short-range interactions in the *NN* $^{1}S_{0}$ channel within $\chi$EFT by a generalization considering the two dibaryon fields with the same quantum numbers. It is intended to reproduce the zero amplitude in this wave in the scattering momentum $k_{0}$ at LO, and to include subleading corrections perturbatively in a way consistent with the RG invariance principle. This leads to a systematic improvement up to NLO where the obtained results fit to empirical phase shifts significantly well up to the pion production threshold^23^. In this new PC, a systematic expansion is developed in the power of $Q/M_{hi}$ with $Q \sim M_{lo}\simeq 100$ MeV, which yields a renormalized amplitude order by order. Since the inverse range of OPE is defined by the pion mass, $m_{\pi }\simeq$ 140 MeV$=\mathscr {O}(M_{lo})$ and its inverse strength is defined by $M_{NN}\simeq$ 290 MeV$=\mathscr {O}(M_{lo})$, thus the OPE interaction emerges at LO^19,20^. Besides, the Coulomb interaction between protons which is contributed to the expansion in powers of the small expansion parameter of $\alpha M_{N}/M_{lo}\sim \aleph /M_{lo}$, with the fine structure constant $\alpha \!\equiv \! e^2/4\pi \!\sim \!1/137$ and $\aleph =\mathscr {O}( M_{lo}^{2}/M_{hi})$, inserts at NLO^23,24^.

This paper is organized as follows: In Section “Pionful EFT of the *NN* systems in new power counting” a reformulation of the $\chi$EFT is provided for the spin singlet channel $^{1}S_{0}$, of *pp* scattering based on a new PC. Then we calculate the *pp* amplitude and phase shift up to NLO. In Section “Conclusion” we summarize our study.

## Pionful EFT of the *NN* systems in new power counting

In this section the theory developed in the pionless effective field theory ( EFT) approach based on new PC for the spin singlet channel $^{1}\!S_{0}$ of *pp* scattering in the previous study^24^, is modified to include the pion exchange. The nuclear pionful effective Lagrangian for the *NN* systems can be written in terms of nucleons and pions instead of the underlying particles such as quarks and gluons of QCD^25,26^. In a new rearrangement of short-range interactions in the $^{1}\!S_{0}$ channel within $\chi$EFT, which is generalized by considering two dibaryon fields $\varvec{\phi }_{1,2}$, the effective Lagrangian is rewritten as^23^

1 $$\begin{aligned} \mathscr {L}_\mathcal {\chi }^{(2\phi )}= & {} \frac{1}{2}\Big (\partial _{\mu }\varvec{\pi }\cdot \partial ^{\mu }\varvec{\pi }-m_{\pi }^{2}\pi ^{2}\Big )+N^{\dagger }\Big [i\partial _{0}+\frac{\nabla ^{2}}{2m_{N}}-\frac{g_{A}}{2f_{\pi }}\varvec{\tau }\cdot \Big (\varvec{\sigma }\cdot \varvec{\nabla }\varvec{\pi }\Big )\Big ]N\nonumber \\{} & {} +\sum _{j=1,2}\Bigg \{\varvec{\phi }^{\dagger }_{j}\cdot \Big [{\Delta }_{j}+c_{j}\Big (i\partial _{0}+\frac{\nabla ^{2}}{4m_{N}}\Big )\Big ]\varvec{\phi }_{j}-\sqrt{\frac{4\pi }{m_{N}}}\Big (\varvec{\phi }^{\dagger }_{j}\cdot N^{T}\varvec{P}_{^{1}S_{0}}N+{\textrm{h}}.{\textrm{c}}.\Big )\Bigg \}+\dots , \end{aligned}$$

where *N* is the isodoublet, bispinor nucleon field, $\varvec{\pi }$ is the isotriplet pion field, $m_{\pi }\!\sim \! Q\! =\!\mathscr {O}(M_{lo})$ is pion mass, $m_{N}\!=\!\mathscr {O}(M_{QCD})$ is the nucleon mass, $g_{A}\!=\!\mathscr {O}(1)$ is the axial vector coupling constant, $f_{\pi }\!=\!\mathscr {O}\big (M_{QCD}/4\pi \big )\!\simeq \!93$ MeV is the pion decay constant, ${\Delta }_{j}$ are the dibaryon residual masses, $c_{j}$ are the dimensionless factors for the kinetic dibaryon terms and $\sqrt{4\pi /m_{N}}$ is the $\varvec{\phi }_{j}NN$ coupling. The projector $\varvec{P}_{^{1}\!S_{0}}$ is expressed in terms of the Pauli matrices $\varvec{\sigma }(\varvec{\tau })$ acting on the spin (isospin) space as

2 $$\begin{aligned} \varvec{P}_{^{1}\!S_{0}(np)}=\sigma _{2}\varvec{\tau }\tau _{2}/\sqrt{8},\quad \quad \varvec{P}_{^{1}\!S_{0}(pp)}=\frac{\sigma _{2}\varvec{\tau }}{\sqrt{16}}\Big (\tau _{1}-i\tau _{2}), \end{aligned}$$

for the neutron–proton (*np*) and *pp* configurations. In the proposed reformulation of the pionful EFT the OPE interaction is involved at LO non-perturbatively while the multiple-pion exchanges are suppressed by powers of the small expansion parameter $(M_{lo}/M_{QCD})^{2}$ as the subleading corrections^23^.

### Pure Coulomb amplitude

The strength of the Coulomb–photon exchanges is provided by the dimensionless Sommerfeld parameter which for the *pp* interaction is written as

3 $$\begin{aligned} \eta =\frac{k_C}{k}=\frac{Z^2\alpha \,\mu }{k}. \end{aligned}$$

**Figure 1:**
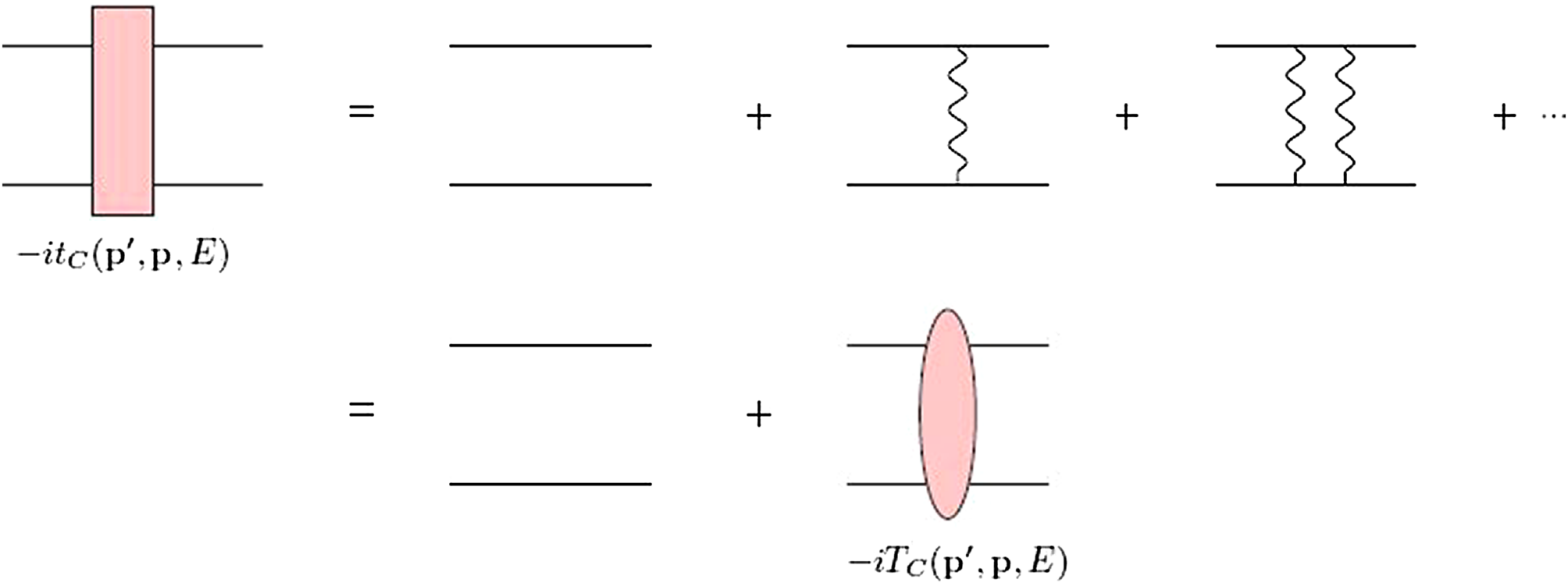
The Coulomb ladder diagrams. The wavy line represent the exchanged photons.

Here $k_C$ is the inverse of Bohr radius of the *pp* system, *k* is the relative momentum of two particles in the CM framework, *Z* indicates the atomic number of proton and $\mu$ denotes the reduced mass of *pp* system. Since each photon-exchange insertion is proportional to $\eta$ so, in the low-energy scattering region, $p\lesssim k_C$, we should consider the full Coulomb interaction non-perturbatively. In order to consider the Coulomb contribution in the *pp* system, we use the Coulomb Green’s function as follows. According to Fig. 1, the Coulomb Green’s function is related to the free Green’s function through the integral equation as^27^

4 $$\begin{aligned} G^{\pm }_C=G^{\pm }_0+G^{\pm }_0\,V_C\,G^{\pm }_C, \end{aligned}$$

where the free and Coulomb Green’s functions for the *pp* system are given by

5 $$\begin{aligned} G^{\pm }_0=\frac{1}{E-H_0\pm i\epsilon },\quad \quad G^{\pm }_C=\frac{1}{E-H_0-V_C\pm i\epsilon }, \end{aligned}$$

with $V_C\!=\!\alpha /r$ and $H_0\!=\!k^2/2\mu$ as the repulsive Coulomb potential between two protons and the free-particle Hamiltonian, respectively. The incoming/outgoing Coulomb states of *pp* system are written as

6 $$\begin{aligned} |\Psi ^{(\pm )}_{\textbf{p}}\rangle =(1+G^{\pm }_{C}\,V_{C})\left| \textbf{p}\right\rangle =(1+G^{\pm }_{0}\,T_{C})\left| \textbf{p}\right\rangle . \end{aligned}$$

So, the pure Coulomb scattering amplitude with considering multiple photon exchanges contribution according to the summation of Coulomb ladder diagrams of Fig. 1 in the partial wave expansion can be written as^27^,

7 $$\begin{aligned} T_C({\textbf {p}}',{\textbf {p}};E)=\langle \textbf{p}'|V_C|\Psi _{\textbf{p}}^{(+)}\rangle = \sum _{l=0}^{\infty } (2l+1) T_C^{l} \,\,P_l(\hat{\textbf{p}}'\cdot \hat{\textbf{p}})=-\frac{2 \pi }{\mu } \sum _{l=0}^{\infty } (2l+1) \frac{e^{2i\sigma _l}-1}{2ik}P_l(\hat{\textbf{p}}'\cdot \hat{\textbf{p}}), \end{aligned}$$

where $k=|\textbf{p}|=|\textbf{p}'|$ and $\sigma _{l}=\mathrm {\arg }\,\Gamma (1+l+i\eta )$ is the Coulomb phase shift. This is the well-known Mott scattering amplitude which holds at very low energies.

### $^{1}\!S_{0}$ channel at LO

The zero amplitude corresponding to the scattering momentum $k_0\simeq 340$ MeV$=\mathscr {O}(M_{lo})$ in the $^{1}\!S_{0}$ channel for the *np* scattering in the new pionful theory PC has been reproduced around the unitarity limit at LO^23^. The Coulomb interaction between protons inters at NLO in this PC scheme^24,28^. The *S*-wave Coulomb-modified effective range expansion (ERE) in terms of the phase shift is given by^29^

8 $$\begin{aligned} C_{\eta }^{2}\,k\,\big ( \textrm{cot}\, \delta _{pp}(k)-i\big )+\alpha \, m_{N}\,H(\eta )=-\frac{1}{a_{C}}+\frac{1}{2}r_{C}\,k^{2}+\frac{1}{4}P_{C}\,k^{4} +\cdots \!, \end{aligned}$$

where $k\!=\!\sqrt{m_{N}E}$, with *E* the CM energy of *pp* system. $C_{\eta }^{2}=2\pi \eta /(\textrm{exp}(2\pi \eta )-1)$ is the probability of finding two interacting charge particles at zero separation and the function *H* is expressed in terms of the digamma function $\psi =(d/dz)\ln {\Gamma }(z)$ as

9 $$\begin{aligned} H(\eta )=\psi (i\eta )+\frac{1}{2i\eta }-\ln (i\eta ). \end{aligned}$$

In Eq. (8) $a_{C}\simeq 7.81$ fm^30^ is the Coulomb-modified scattering length, $r_{C}\simeq 2.7$ fm^31^ indicates the Coulomb-modified effective range and $P_{C}\simeq 2.0$ fm$^{3}$^32^ denotes the Coulomb-modified shape parameter. Since $1/|a_{C}|=\mathscr {O}(M_{lo}^{2}/M_{hi})$ is small in comparison with the typical nuclear scale and is suppressed by the power of the breakdown scale $M_{hi}$, thus we remain close to the unitary limit in $^{1}\!S_{0}$ channel. Therefore as *np* system, we can take $r_{C}=r_{0}\simeq 2.7$ fm and $P_{C}=P_{0}\simeq 2.0$ fm$^{3}$ for *pp* scattering at LO.

The LO potential for the *np* and *pp* systems is the sum of the dibaryon arrangement of short-range potential $V_{S}^{[0]}$, inspired by the  EFT and the long-range spin singlet projection of OPE potential $V_{L}^{[0]}$ as^23^

10 $$\begin{aligned} V^{[0]}(\textbf{p}^{\prime },\textbf{p},k;{\Lambda })\equiv & {} V^{[0]}_{S}(k;{\Lambda })+V^{[0]}_{L}(\textbf{p}^{\prime },\textbf{p}) \nonumber \\= & {} \frac{4\pi }{m_N}\Big [\frac{1}{{\Delta }^{[0]}_{1}({\Lambda })}+\frac{1}{{\Delta }^{[0]}_{2}({\Lambda })+c^{[0]}_{2}({\Lambda })\,k^{2}/m_{N}}\Big ] -\frac{4\pi }{m_N}\frac{m_{\pi }^{2}}{M_{NN}}\frac{1}{(\textbf{p}^{\prime }-\textbf{p})^{2}+m^{2}_{\pi }}, \end{aligned}$$

where the superscript $^{[i]}$ indicates the order of the quantities. Here, $\textbf{p}\, (\textbf{p}')$ is the relative incoming (outgoing) momentum of two nucleons and ${\Lambda }$ is a momentum cutoff in the range of ${\Lambda }\ge \! M_{hi}\!\gg k$ which is regularized the loop integrals. $V_{L}^{[0]}$ is the Yukawa potential with the inverse OPE strength $M_{NN}=16\pi f_{\pi }^{2}/g_{A}^{2}m_{N} \approx 290~ \textrm{ MeV}$^19,20^. Moreover, the momentum-independent contact piece of OPE has been absorbed in the short-range potential via the redefinition

11 $$\begin{aligned} \Bigg (\frac{1}{{\Delta }_{1}^{[0]}({\Lambda })}+\frac{1}{M_{NN}}\Bigg )^{-1}\rightarrow {\Delta }_{1}^{[0]}({\Lambda }). \end{aligned}$$

The LO off-shell *pp* scattering amplitude is obtained by solving the LS equation using the LO potential of Eq. (10)

12 $$\begin{aligned} T^{[0]}(\textbf{p}^{\prime },\textbf{p},k;{\Lambda })=V^{[0]}(\textbf{p}^{\prime },\textbf{p},k;{\Lambda })-m_N\!\!\int \frac{d^{3}q}{(2\pi )^{3}}\frac{f(q/{\Lambda })}{q^{2}-k^{2}-i\epsilon }V^{[0]}(\textbf{p}^{\prime },\textbf{q},k;{\Lambda })\,T^{[0]}(\textbf{q},\textbf{p},k;{\Lambda }), \end{aligned}$$

where $f(q/{\Lambda })$ is a regulator function for the loop integrals. For example, this is equal with a step function $f(q/{\Lambda })\!=\!\theta \big (1-q/{\Lambda }\big )$, for a sharp cutoff prescription which we use in the numerical calculations. Projection of Eq. (12) in to the *S*-wave leads to

13 $$\begin{aligned} T^{[0]}_{0}({p}^{\prime },{p},k;{\Lambda })=V^{[0]}_{0}({p}^{\prime },{p},k;{\Lambda })-m_N\!\!\int _{0}^{{\Lambda }}\frac{dq \,q^{2}}{(2\pi )^3}\,\frac{V^{[0]}_{0}({p}^{\prime },{q},k;{\Lambda })}{q^2-k^2-i\epsilon }\,T^{[0]}_{0}({q},{p},k;{\Lambda }), \end{aligned}$$

with

14 $$\begin{aligned} V^{[0]}_{0}({p}^{\prime },{p},k;{\Lambda })\!=\! 2\pi \int _{-1}^{1} dx \, V^{[0]}({p}^{\prime },{p},x,k;{\Lambda }),\end{aligned}$$

15 $$\begin{aligned} T^{[0]}_{0}({p}^{\prime },{p},k;{\Lambda })\!=\! 2\pi \int _{-1}^{1} dx\, T^{[0]}({p}^{\prime },{p},x,k;{\Lambda }), \end{aligned}$$

where $x=\hat{\textbf{p}}'\cdot \hat{\textbf{p}}$. According to Eq. (14) the *S*-wave projection of the potential of Eq. (10) is written as

16 $$\begin{aligned} V_{0}^{[0]}(p^{\prime },p,k,{\Lambda })\! &=\! \frac{(4\pi )^2}{m_N}\Bigg (\frac{1}{{\Delta }^{[0]}_{1}({\Lambda })}+\frac{1}{{\Delta }^{[0]}_{2}({\Lambda })+c^{[0]}_{2}({\Lambda })\,k^{2}/m_{N}}\Bigg )-\frac{8\pi ^2 m_{\pi }^{2}}{m_N M_{NN}}\int _{-1}^{1}\frac{dx}{p^{\prime 2}-p^{2}-2pp^{\prime }x+m_{\pi }^{2}} \nonumber \\ \! &=\! \frac{(4\pi )^2}{m_N}\Bigg (\frac{1}{{\Delta }^{[0]}_{1}({\Lambda })}+\frac{1}{{\Delta }^{[0]}_{2}({\Lambda })+c^{[0]}_{2}({\Lambda })\,k^{2}/m_{N}}\Bigg )-\frac{8\pi ^2}{pp'}\frac{m_{\pi }^{2}}{m_N M_{NN}}\,\,Q_{0}\Bigg (\frac{p^2+p^{\prime 2}+m^{2}_{\pi }}{2pp'}\Bigg ),\; \end{aligned}$$

where $Q_0(x)\!=\!\frac{1}{2}\,\textrm{ln}(\frac{x+1}{x-1})$ is the second kind of Legendre function with $l\!=\!0$. We have solved Eq. (13) for the $^{1}\!S_{0}$ channel numerically as done in Refs.^14,23^. To this end, the infrared singularity at $q\!=\!k$ is separated into Cauchy’s principal value and a delta function. Then the principal value singularity is treated by standard subtraction technique^14^. Finally, the integral equation (13) is solved by Gauss-Legendre quadrature method^14^. The two dibaryon parameters ${\Delta _{1}^{[0]}}$ and $\mathrm {c_{2}^{[0]}}$ in the $V_{S}^{[0]}$ , which are needed to describe the amplitude zero and its energy dependence near threshold and third parameter ${\Delta _{2}^{[0]}}$ which ensures the fine tuning that leads to a large scattering length, are sufficient for renormalization of residual cutoff dependency. These three cutoff-dependent bare parameters are determined by imposing three cutoff-independent conditions on the on-shell *pp* amplitude of Eq. (13) as

- Unitarity limit, $$1/a_C^{[0]}=0$$;
- Physical effective range, $r_{C}=2.7$ fm;
- Physical amplitude zero, $k_{0}=340.4$ MeV.

The values of the three dibaryon parameters through numerical calculation must be very well tuned in order to reproduce the experimental data, $1/a_C^{[0]}$, $r_{C}$ and $k_{0}$. In order to simplify the numerical solution of the LS equation we take the scattering length large and negative, $a_{0}^{[0]}\!=\!\!-600$ fm^23^. Finally, the LO pionful phase shift for the $^{1}\!S_{0}$ channel is obtained from

17 $$\begin{aligned} \delta ^{[0]}(k;{\Lambda })=\mathrm {-cot^{-1}}\Bigg (\frac{4\pi }{m_{N}k}\textrm{Re}\Big (T_{0}^{[0]}(k,k,k;{\Lambda })\Big )^{-1} \Bigg ).\quad \quad \end{aligned}$$

**Figure 2:**
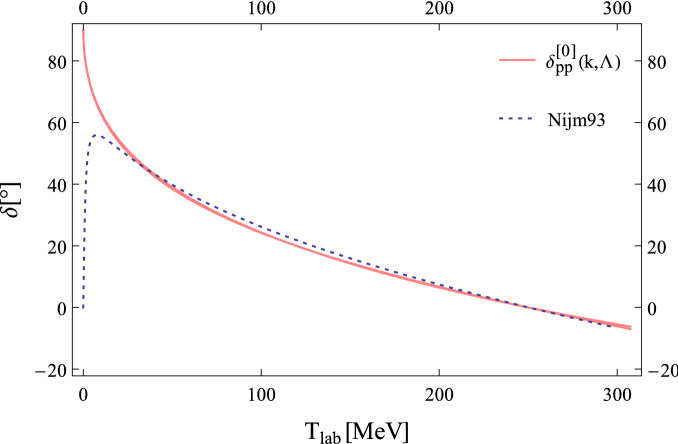
The *pp* $^{1}\!S_{0}$ phase shift against laboratory energy $T_{lab}=2k^{2}/m_{N}$ for $\chi$EFT at LO in new PC . The color narrow band shows the evolution of the sharp cutoff from 600 MeV to 2 GeV, The dashed line indicates the result obtained from the Nijm93 potential model^22^.

Since the Coulomb-modified scattering length $a^{[0]}_{C}$, is still large compared to the typical nuclear length scale set by the inverse $m_{\pi }$ in the pionful EFT, so we remain close to unitarity limit in the $^{1}\!S_{0}$ *pp* channel. Thus the $^{1}\!S_{0}$ *pp* phase shift obtained from on-shell *S*-wave projected *T*-matrix at LO now behaves like the $^{1}\!S_{0}$ *np* phase shift at this order. We present the LO pionful obtained result for the $^{1}\!S_{0}$ *pp* phase shift for various cutoffs from 600 MeV to 2 GeV in Fig. 2 and compare to the results obtained from the Nijm93 potential model^22^. As it shown in Fig. 2 the cutoff dependence of $\delta ^{[0]}$ is little while the cutoff ${\Lambda }$ varies from 600 MeV to 2 GeV.

### $^{1}S_{0}$ channel up to NLO

In this section, we follow the same procedure as for the $^{1}\!S_{0}$ channel of *np* scattering in order to consider for *pp* scattering up to NLO in the presence of Coulomb interaction. In the new PC two-pion exchange enters at $\mathrm {N^{2}LO}$ and the higher orders. Moreover, the Coulomb interaction between protons contributes an expansion in $\alpha m_{N}/M_{lo}\sim \mathscr {O}(M_{lo}/M_{hi})$^23^. So, we should consider the Coulomb interaction at NLO. The *pp* short-range potential at NLO is given by^23^

18 $$\begin{aligned} V_{S}^{[1]}(k;{\Lambda })\!=\! & {} -\frac{4\pi }{m_{N}}\sum _{j}\Bigg ({\Delta }_{j}^{[0]}({\Lambda })+c_{j}^{[0]} ({\Lambda })\,\frac{k^{2}}{m_{N}}\Bigg )^{-2} \Bigg ({\Delta }_{j}^{[1]}({\Lambda })+c_{j}^{[1]}({\Lambda }) \,\frac{k^{2}}{m_{N}}\Bigg )\quad \nonumber \\\!=\! & {} -\frac{4\pi }{m_{N}}\Bigg[\frac{{\Delta _{1}^{[1]}(\Lambda )}+c^{[1]}_{1}({\Lambda })\frac{k^{2}}{m_{N}}}{{\Delta ^{[0]2}_{1}(\Lambda )}} +\frac{{\Delta _{2}^{[1]}(\Lambda )}+c^{[1]}_{2}({\Lambda })\frac{k^{2}}{m_{N}}}{\bigg ({\Delta ^{[0]}_{2}(\Lambda )}+c_{2}^{[0]} ({\Lambda })\frac{k^{2}}{m_{N}}\bigg )^{2}}\Bigg], \end{aligned}$$

where the LO dibaryon parameters are obtained by fine tuning in the previous subsection. Using the LS equation, one is able to express the full NLO scattering amplitude $T^{[1]}$ in terms of multiple insertions of the operator $G^{\pm }_{SL}\,(V_S^{[1]}+V_C)$ acting on the LO *pp* scattering state $|\chi _\textbf{p}^{[0]}\rangle$ as

19 $$\begin{aligned} T^{[1]}=\sum _{n=0}^{\infty }\langle \chi ^{[0]}_{\textbf{p}'}|(V_S^{[1]}+V_C)\,\big [G^{\pm }_{SL}\,(V_S^{[1]}+V_C)\big ]^n |\chi ^{[0]}_\textbf{p}\rangle , \end{aligned}$$

with the full incoming/outgoing *pp* state in terms of the full LO amplitude

20 $$\begin{aligned}{} & {} |\chi ^{[0]}_\textbf{p}\rangle =(1+G^{\pm }_{SL} V^{[0]})\left| \textbf{p}\right\rangle =(1+G^{\pm }_{0} T^{[0]})\left| \textbf{p}\right\rangle , \end{aligned}$$

21 $$\begin{aligned}{} & {} G^{\pm }_{SL}=G^{\pm }_0+G^{\pm }_0\,V^{[0]}\,G^{\pm }_{SL}, \end{aligned}$$

where the free and $G_{SL}$ Green’s functions are given by

22 $$\begin{aligned} G^{\pm }_0=\frac{1}{E-H_0\pm i\epsilon },\quad \quad G^{\pm }_{SL}=\frac{1}{E-H_0-V^{[0]}\pm i\epsilon }. \end{aligned}$$

According to the new PC short-range interaction $V_{S}^{[1]}$, treats perturbatively. Moreover, the Coulomb interaction for the momentum scattering region $p>k_C\approx 3.42$ MeV, corresponding to the laboratory energy $T_{lab}=0.025$ MeV, treats perturbatively. Thus, based on distorted-wave perturbation theory we can consider the first term of born series $(n=0)$ in Eq. (19) in calculations. In the low-energy region $(T_{lab}\lesssim 0.025 \,\textrm{MeV})$, we can use the results obtained from  EFT theory with considering Coulomb interaction non-perturbatively as is done in the previous study^24^. Consequently, Eq. (19) is reduced to

23 $$\begin{aligned} T^{[1]}=\langle \chi ^{[0]}_{\textbf{p}'}|\,(V_S^{[1]}+V_C)\ |\chi ^{[0]}_\textbf{p}\rangle . \end{aligned}$$

Due to the presence of Coulomb potential, we have numerical challenge for calculation of NLO *pp* amplitude $T^{[1]}$. This challenge is due to the singularities that occurring in the on-shell momentums $p=p'$. To avoid this difficulty we consider some other terms from series of Eq. (19), corresponding to the diagrams in Fig. 1, in Eq. (23) as

24 $$\begin{aligned} T^{[1]}=\langle \chi ^{[0]}_{\textbf{p}'}|\,(V_S^{[1]}+V_C+\sum _{n=1}^{\infty }V_C(G^{\pm }_0V_C)^n\ |\chi ^{[0]}_\textbf{p}\rangle , \end{aligned}$$

So, we can rewrite Eq. (24) as

25 $$\begin{aligned} T^{[1]}=\langle \chi ^{[0]}_{\textbf{p}'}|\,(V_S^{[1]}+T_C)\ |\chi ^{[0]}_\textbf{p}\rangle . \end{aligned}$$

Finally, we obtain the separable NLO chiral *pp* amplitude using the insertions of short- and long-range interactions together with Coulomb interaction as

26 $$\begin{aligned} T^{[1]}(\textbf{p}',\textbf{p},k;{\Lambda })=\chi ^{[0]}(\textbf{p}',k;{\Lambda })\Big [V^{[1]}_{S}(k;{\Lambda })+T_{C}(\mathbf {p^{\prime }},\textbf{p},E)\Big ]\chi ^{[0]}(\textbf{p}',k;{\Lambda }), \end{aligned}$$

where

27 $$\begin{aligned} \chi ^{[0]}(\textbf{p},k;{\Lambda })=1-m_N\!\!\int \!\!\frac{d^{3}q}{(2\pi )^{3}}\frac{f(q/{\Lambda })}{q^{2}-k^{2}-i\epsilon }T^{[0]}(\textbf{p},\textbf{q},k;{\Lambda }). \end{aligned}$$

Equation (26) represents the total elastic scattering amplitude which is included the pure Coulomb amplitude. Subtracting the pure Coulomb amplitude from Eq. (26) yields

28 $$\begin{aligned} T^{[1]}(\textbf{p}',\textbf{p},k;{\Lambda })=\chi ^{[0]}(\textbf{p}',k;{\Lambda })\Big [V^{[1]}_{S}(k;{\Lambda })+T_{C}(\mathbf {p^{\prime }},\textbf{p},E)\Big ]\chi ^{[0]}(\textbf{p}',k;{\Lambda })-T_{C}(\mathbf {p^{\prime }},\textbf{p},E), \end{aligned}$$

Projection of Eq. (28) into the *S*-wave leads to

29 $$\begin{aligned} T_{0}^{[1]}(p',p,k;{\Lambda })=\chi _{0}^{[0]}(p',k;{\Lambda })\,\Big [V^{[1]}_{S}(k;{\Lambda })+T^0_{\;C}(p^{\prime },p,E)\Big ]\chi _{0}^{[0]}(p,k;{\Lambda })-T^{0}_{\;C}(p^{\prime },p,E), \end{aligned}$$

with

30 $$\begin{aligned} \chi _{0}^{[0]}(p,k;{\Lambda })=1-m_N\int _{0}^{{\Lambda }}\frac{dq\,q^2}{(2\pi )^{3}}\,\frac{T_{0}^{[0]}(p,q,k;{\Lambda })}{q^{2}-k^{2}-i\epsilon }, \end{aligned}$$

and

31 $$\begin{aligned} T^{0}_{\;C}(p^{\prime },p,E)=-\frac{4\pi }{m_{N}}\frac{e^{2i\sigma _{0}}-1}{2ik},\quad \quad e^{2i\sigma _{0}}=\Gamma (1+i\eta )/\Gamma (1-i\eta ). \end{aligned}$$

**Figure 3:**
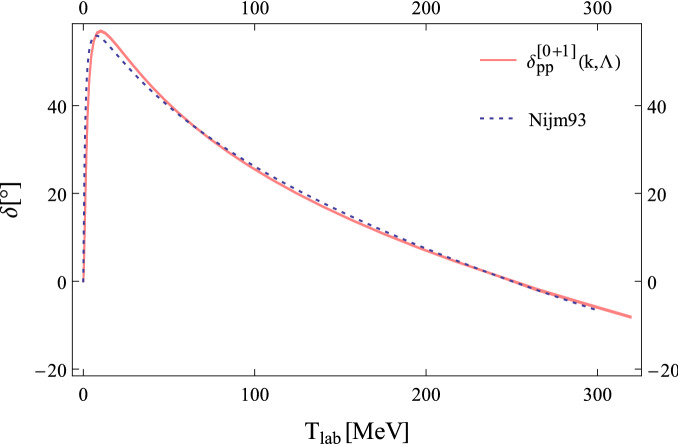
The *pp* $^{1}\!S_{0}$ phase shift against laboratory energy $T_{lab}=2k^{2}/m_{N}$ for $\chi$EFT in new the PC up to NLO. The color narrow band shows the evolution of the cutoff from 600 MeV to 2 GeV. The dotted line indicates the result obtained from Nijm93 potential model.^22^.

For calculation of the on-shell *S*-wave amplitude at NLO, as a first step we should obtain $\chi _{0}^{[0]}(p,k;{\Lambda })$ from Eq. (30). This is done by discretizing the integral and momentum variables by Gauss–Legendre quadrature mesh points. Then the singularity of the integral kernel is handled by Cauchy’s principal-value formula and Cauchy’s principal-value is computed by standard subtraction technique^14^. In the second step, we calculate the *S*-wave Coulomb corrections from Eq. (31). Finally, the pionful *pp* phase shift up to NLO is obtained from

32 $$\begin{aligned} \delta ^{[0+1]}(k;{\Lambda })=- \textrm{cot}^{-1}\Bigg (\frac{4\pi }{m_{N}k}\textrm{Re}\Bigg [\Big (T^{[0]}_{0}(k,k,k;{\Lambda })\Big )^{-1}\,\Bigg (1-\frac{T^{[1]}_{0}(k,k,k;{\Lambda })}{T^{[0]}_{0}(k,k,k;{\Lambda })}\Bigg )\Bigg ]\Bigg ). \end{aligned}$$

The dibaryon parameters are fixed by imposing the four cutoff independent conditions which are the values of *pp* phase shifts at four corresponding momenta obtained from Nijm93 potential model^22^:

- $$\delta ^{[0+1]}(20.5$$ $$\textrm{MeV};{\Lambda })=30.5^{\circ };$$
- $$\delta ^{[0+1]}(41.1$$ $$\textrm{MeV};{\Lambda })=52.9^{\circ };$$
- $$\delta ^{[0+1]}(238.0$$ $$\textrm{MeV};{\Lambda })=21.8^{\circ };$$
- $$\delta ^{[0+1]}(342.6$$ $$\textrm{MeV};{\Lambda })=0^{\circ }.$$

The pionful LO + NLO phase shifts are shown in Fig. 3, the narrow band shows the evolution of the cutoff parameter from 600 MeV to 2 GeV. By increasing the cutoff the fine tuning of dibaryon parameters becomes more and more noticeable. As can be seen the cutoff dependence of *pp* phase shift is improved well up to the pion production threshold.

## Conclusion

In this paper, we have studied the *pp* scattering within $\chi$EFT based on new power counting. To this end, we have considered a new rearrangement of the short-range interactions in the $^{1}S_{0}$ *pp* channel in the presence of Coulomb interaction in a way consistent with RG invariance. This leads to the existence of the zero amplitude at LO in scattering momentum about 340 MeV. We calculate NLO corrections and show a systematic improvement in the description of the phase shift.

Moreover, the empirical phase shift for the *pp* singlet channel fit remarkably well up to the pion production threshold. The LO+NLO results obtained within $\chi$EFT from the on-shell *S*-wave projected two-body *T*-matrix through numerical calculation of LS equation have little cutoff dependence even though ${\Lambda }$ is changed from 600 MeV to 2 GeV. To improve the results, we intend the *pp* scattering part to investigate the contribution of higher orders however, it is anticipated to provide only very small corrections in distorted-wave perturbation theory.

## Data Availability

The datasets used and/or analysed during the current study available from the corresponding author on reasonable request.
